# Supplementary material for: Socioeconomic inequalities in overweight and obesity among adults: Results from the Indonesian Food Barometer study
Source: PLoS One. 2025 Dec 5;20(12):e0337170. doi: 10.1371/journal.pone.0337170 (PMC12680161; doi:10.1371/journal.pone.0337170)
Supplement: S1 Table — The table describes the completeness of data and sample exclusions in the Indonesian Food Barometer dataset. (DOCX) [file pone.0337170.s001.docx]

**S1 Table.** Sample characteristics and data completeness

| **Variables** | **Complete cases** | | **Missing** | **Note** |
| --- | --- | --- | --- | --- |
|  | **n** | **%** | **n (%)** |  |
| Initial dataset | 1772 |  |  |  |
| Sample after exclusion of pregnant women (n=67) | 1660 |  |  |  |
| Sample after removing missing strata | 1610 |  | 50 (3.0) | Missing data on strata (province and urban/rural) |
| Overweight or obesity |  |  | 2 (0.1) | Missing data on height |
| Yes | 896 | 56.4 |  |  |
| No | 712 | 43.6 |  |  |
| Sex |  |  | 0 (0.0) |  |
| Male | 847 | 53.0 |  |  |
| Female | 763 | 47.0 |  |  |
| Age group |  |  | 0 (0.0) |  |
| 18-25 | 339 | 21.4 |  |  |
| 26-35 | 449 | 27.9 |  |  |
| 36-45 | 321 | 20.6 |  |  |
| ≥46 | 501 | 30.1 |  |  |
| Education level |  |  | 0 (0.0) |  |
| Primary or lower | 666 | 45.3 |  |  |
| Secondary | 702 | 43.6 |  |  |
| Higher | 242 | 11.1 |  |  |
| Occupation |  |  | 3 (0.04)^1^ |  |
| Professional | 54 | 2.5 |  |  |
| White-collar | 508 | 30.5 |  |  |
| Blue-collar | 434 | 26.3 |  |  |
| Not working^2^ | 611 | 40.7 |  |  |
| Marital status |  |  | 0 (0.0) |  |
| Single | 380 | 22.5 |  |  |
| Married | 1113 | 68.9 |  |  |
| Widowed/ divorced | 117 | 8.6 |  |  |
| Have a child |  |  | 0 (0.0) |  |
| Yes | 1175 | 74.5 |  |  |
| No | 435 | 25.5 |  |  |
| Wealth status |  |  | 3 (0.01)^1^ |  |
| Q1 (poorest) | 220 | 20.3 |  |  |
| Q2 | 299 | 20.1 |  |  |
| Q3 | 320 | 19.5 |  |  |
| Q4 | 387 | 20.2 |  |  |
| Q5 (richest) | 381 |  |  |  |
| Religion |  |  | 0 (0.0) |  |
| Muslim | 1260 | 92.3 |  |  |
| Others^3^ | 350 | 7.7 |  |  |
| Place of residence |  |  | 0 (0.0) |  |
| Urban | 973 | 66.9 |  |  |
| Rural | 637 | 33.1 |  |  |
| Province |  |  | 0 (0.0) |  |
| West Sumatera | 257 | 5.3 |  |  |
| DKI Jakarta | 265 | 9.4 |  |  |
| West Java | 277 | 42.2 |  |  |
| East Java | 265 | 31.1 |  |  |
| Balinese | 279 | 4.4 |  |  |
| South Sulawesi | 267 | 7.6 |  |  |

^1^ Percentages of missing data are calculated based on the total relevant sample for each variable. Differences despite the same n may be due to denominators or weighting.

^2^ Housewives, student, and not working

^3^ Christian, Catholic, Hinduism, Buddhist, Confucianism

The sample characteristics in Table S1 are based on respondents remaining after excluding pregnant women and those with missing strata information (n = 1,610). The final analytic sample (n = 1,604) further excludes cases with missing data on analysis variables, and its characteristics are presented in Table 1. All estimates and percentages are weighted and adjusted for the complex survey design, including stratification by province and urban/rural residence, primary sampling units, and sampling weights.
