# Supplementary material for: Socioeconomic inequalities in overweight and obesity among adults: Results from the Indonesian Food Barometer study
Source: PLoS One. 2025 Dec 5;20(12):e0337170. doi: 10.1371/journal.pone.0337170 (PMC12680161; doi:10.1371/journal.pone.0337170)
Supplement: Attachment — Submitted filename: Response to Reviewers.docx [file pone.0337170.s003.docx]

| Comments | Response  [Pages and lines refer to the file ‘Revised Manuscript with Track Changes’] |
| --- | --- |
| **Journal requirements** |  |
| 1. When submitting your revision, we need you to address these additional requirements.  Please ensure that your manuscript meets PLOS ONE's style requirements, including those for file naming. The PLOS ONE style templates can be found at https://journals.plos.org/plosone/s/file?id=wjVg/PLOSOne_formatting_sample_main_body.pdf and https://journals.plos.org/plosone/s/file?id=ba62/PLOSOne_formatting_sample_title_authors_affiliations.pdf | We have checked and revised the manuscript according to the requirements. |
| 2. In the online submission form, you indicated that [Public availability of the dataset from the 2018 Indonesian Food Barometer by SEAMEO RECFON based on Open Science philosophy is in progress. Raw data sets are available on request from the corresponding author.].  All PLOS journals now require all data underlying the findings described in their manuscript to be freely available to other researchers, either 1. In a public repository, 2. Within the manuscript itself, or 3. Uploaded as supplementary information.  This policy applies to all data except where public deposition would breach compliance with the protocol approved by your research ethics board. If your data cannot be made publicly available for ethical or legal reasons (e.g., public availability would compromise patient privacy), please explain your reasons on resubmission and your exemption request will be escalated for approval.  When completing the data availability statement of the submission form, you indicated that you will make your data available on acceptance. We strongly recommend all authors decide on a data sharing plan before acceptance, as the process can be lengthy and hold up publication timelines. Please note that, though access restrictions are acceptable now, your entire data will need to be made freely accessible if your manuscript is accepted for publication. This policy applies to all data except where public deposition would breach compliance with the protocol approved by your research ethics board. If you are unable to adhere to our open data policy, please kindly revise your statement to explain your reasoning and we will seek the editor's input on an exemption. Please be assured that, once you have provided your new statement, the assessment of your exemption will not hold up the peer review process. | Many thanks for the comment on this. We have revised the statement according to the situation here based on the existing regulations by the Indonesian Ministry of Health Regulations (*Permenkes*) No. 85/2020 which pertains to the Transfer and Use of Materials, Information Content, and Data. Thus, we were unable to make the dataset publicly available, but it can be requested to the research unit of the SEAMEO RECFON.  ”Data is not publicly available, but requests for the data underlying the study can be directed to the Research Unit of the SEAMEO RECFON at research@seameo-recfon.org” |
| 3. Your ethics statement should only appear in the Methods section of your manuscript. If your ethics statement is written in any section besides the Methods, please move it to the Methods section and delete it from any other section. Please ensure that your ethics statement is included in your manuscript, as the ethics statement entered into the online submission form will not be published alongside your manuscript. | Thank for the comment. We already included the ethics statement in the Methods section only. Page 8 Lines 187-190 |
| **Reviewer 1**  The manuscript presents an important and timely study investigating the socioeconomic inequalities in overweight and obesity among Indonesian adults, based on data from the Indonesian Food Barometer. As the prevalence of overweight and obesity in Indonesia continues to rise, particularly across different socioeconomic strata, this study provides valuable insights into the factors contributing to this trend. The authors present a comprehensive analysis of how wealth status, education levels, and place of residence contribute to the observed inequalities in overweight and obesity in Indonesia. Given the global importance of addressing the dual burden of malnutrition, this research makes a significant contribution to the existing literature. | Many thanks for your valuable inputs. We have revised the manuscripts accordingly as indicated below: |
| Specific Suggestions: |  |
| *Introduction*:   - The introduction is well-written and clearly sets the stage for the study. However, I recommend further elaborating on the concept of the "nutrition transition" in Indonesia. While the manuscript touches on this, a bit more background on how this transition has influenced both undernutrition and overnutrition could provide greater context for the study's findings. - It would be helpful to briefly mention the existing literature on the relationship between socioeconomic status and obesity in other countries, as this would position the Indonesian findings in a broader international context. | - Thank you for the suggestion, we have added information about nutrition transition in Indonesia. Pages 3-4 Lines 58-67. - The relationship between socioeconomic status and overweight/ obesity has been described in page 3 lines 46-57, focusing on the level of country’s development. We also added new literature on it from several countries in South and Southeast Asia in page 4 lines 79-81. |
| *Methods*:   - The methods section is generally clear, but it would be useful to provide a bit more detail about how the socioeconomic status (SES) was measured. Was it based on income, wealth quintiles, or a combination of factors? A brief description of how SES was categorized would clarify the analysis for readers. - It would also be useful to include more details about how education levels were categorized. Were they grouped by specific attainment levels (e.g., primary, secondary, tertiary), and how were these related to the socioeconomic status stratifications? | - In this study, we use wealth status (or wealth index) and education level as the measures representing socioeconomic status. Explanation on the measurements of both variables is presented in pages 5-6 subchapter “Socioeconomic status”: “The wealth variable was derived from household assets through the application of principal component analysis. The assets encompassed possession of electronic devices, livestock, and various amenities, such as a water source, cooking fuel, and materials for the floor, walls, and roof. The score was determined by utilising the factor score derived from principal component analysis (PCA) with varimax rotation [17]. Subsequently, we categorized these scores into quintiles, ranging from Q1 (representing the 20% of individuals with the lowest scores) to Q5 (representing the 20% of individuals with the highest values).” - Thanks for the suggestion, and we have added more information for the education level: “The education level was categorized into three groups: primary education or below (primary school or no formal education), secondary education (lower and upper secondary school), and higher education (college or university).”   These two measures of socioeconomic status comprises several categories or levels which are regarded as strata from the lowest to the highest level. |
| *Results*:   - The results are well-presented, but there is room for more in-depth analysis of the relationship between place of residence and obesity levels. Are there significant regional disparities in the prevalence of overweight and obesity, particularly between urban and rural areas? - Further exploration of how geographic location might influence dietary patterns, access to healthcare, and lifestyle factors would be valuable. - Additionally, it may be helpful to include some basic demographic information on the sample population, such as age distribution and gender, to allow readers to better understand the characteristics of the sample. | - Many thanks for the suggestion. We have added the analysis on the association between sample characteristics and the outcome that were embedded in Table 1 (Column 4-5) [P8-9]. Description on the related findings were in page 8. The finding indicated that he prevalence of OAO was different by place of residence: “Urban population accounted for around two-thirds of the sample, and they had a higher odds to live with OAO (AOR 1.50; 95% CI 1.02-2.21).” [page 8 lines 204-206] - In this analysis, we did not include variables of dietary patterns, access to healthcare, and lifestyle factors. Therefore, we mentioned these in the Discussion across the paragraphs for example when discussing the role of place resident in page 14-15 lines 395-429. We also mentioned these in the limitations (page 18 lines 543-545). - Information on the demographic characteristics was presented in Table 1, and described in page 8 lines 191-207 together with the % of OAO and the AORs. Thanks for the comment on this. |
| *Discussion*:   - The discussion provides a solid interpretation of the results and contextualizes the findings within the broader literature. However, the authors should consider expanding on the policy implications of the study. Given the increasing prevalence of obesity in Indonesia, what public health interventions might help address the socioeconomic disparities observed in this study? Recommendations for future research could also be included, particularly regarding potential interventions targeting at-risk populations. - While the study highlights the role of education levels and wealth status in driving inequalities, it would be interesting to see more discussion on the role of cultural factors, urbanization, and food environments in shaping obesity trends across different socioeconomic groups. | Many thanks for the comments. We have revised the Discussion section accordingly.   - Regarding policy implications, we added at the end of paragraph after discussing a particular finding, for example in page 14 lines 381-389 after explaining the role of wealth status and education on OAO. We also summarised the policy implications in pages 17-18 lines 511-537. For future research, we integrated it in the paragraph discussing the limitations in pages 18 lines 540-545. - Elaboration on the role of culture, urbanisation and food environment have been integrated when discussing each contributing variable, for example in page 15 lines 436-444 related to cultural norms on females across different levels of SES, and page 17 lines 502-505 about cultural aspects related to marital status. Regarding urbanisation and food environment, we added the explanation in page 14-15 lines 395-429 and page 12 lines 319-325. |
| *Conclusion*:  The conclusion is concise and effectively summarizes the main findings. However, it would be useful to more explicitly state the potential consequences of these inequalities for Indonesia's public health system and what specific actions might be needed to address these growing disparities in obesity prevalence. | Thanks for the input. We have revised the Conclusion by adding the potential consequences towards the health system and particular action. Page 19 lines 556-562. |
| *Figures and Tables:*   - The manuscript would benefit from a clearer presentation of the data in the tables and figures. Specifically, the tables could be more informative if they included effect sizes or confidence intervals for the reported associations, which would give readers a better sense of the magnitude of the relationships observed. - The figures should also be more clearly labeled, with additional information in the legends to help readers fully understand the data being presented. | - Thanks for the suggestions. We have added the effect sizes (adjusted odds ratios) and confidence intervals for results in Table 1. The revisions were also made as you suggested regarding the Results section. Pages 8-9. - We did not include any figure in the submission. If it is related the Tables, the footnotes have been revised accordingly so that the readers can understand the information presented. |
| Minor Technical Corrections:   - In the abstract, the sentence structure could be improved for clarity. For example, instead of saying "The data from the Indonesian Food Barometer represent around a half of Indonesian population," it could be rephrased as "The data from the Indonesian Food Barometer represent approximately half of the Indonesian population." - There are a few instances where the authors use "obesity" and "overweight" without defining the specific cutoffs used to classify individuals in these categories. It would be helpful to include these definitions for the sake of transparency. | - We did not find the sentence “The data from the Indonesian Food Barometer represent around a half of Indonesian population". In the Abstract, we wrote “Data were extracted from 1602 Indonesian adults participated in the 2018 Indonesian Food Barometer study conducted in six provinces of Indonesia.” We just found that the sentence you quoted is in the Abstract in the online submission (not in the Manuscript), which was our mistake as it should be the same as the Abstract in the Manuscript. Many thanks for the correction. - The cut-off for the outcome (overweight and obesity [OAO]) has been added (Page 2 lines 8-9). Thanks for the suggestion. |
| **Reviewer 2**  Thank you for your submission. This manuscript, investigating socioeconomic inequalities in overweight and obesity (OAO) among Indonesian adults, addresses a critical public health issue with the potential for significant impact. However, the current presentation and methodology require substantial revisions to meet the standards for publication in PLOS ONE. While the research topic is highly relevant, several methodological, analytical, and interpretive limitations weaken the study's overall impact. | Thanks very much for taking the time to review the manuscript. We have revised the manuscripts accordingly as shown below: |
| - My primary concern revolves around the **methodological** approach. While the chosen statistical method, the Wagstaff normalized concentration index (WCI), is relevant for analysing socioeconomic inequalities, the manuscript lacks a robust justification for its selection. The authors do not fully explain why the WCI was chosen over other possible methods, and also does not sufficiently discuss the rationale behind using the 'svy' command in Stata, which could influence the robustness of the results. It is vital to provide a more detailed explanation of the methodology to convince the scientific community of the findings. - The cross-sectional study design is also a major concern. While the authors have collected relevant data, making causal statements from cross-sectional data weakens the validity of the conclusions, particularly when discussing the complex interplay between socioeconomic factors and health outcomes. Therefore, it is important for the authors to acknowledge the study's limitations and ensure that the inferences made from the data are accurate. | Many thanks for the suggestions.   - We have added literature on the reason for choosing the WCI [Page 7 lines 157-160] and using ‘svy’ command in Stata [Page 7 Lines 172-173]. - Regarding the study design, we acknowledged the limitations “First, results on the observed inequalities and their contributing factors should be seen as association rather than causality since the data were collected at one point in time.” Page 18 lines 538-540. |
| - Furthermore, the discussion section requires significant strengthening. Some conclusions are made without adequate support from the presented data, and the interpretations are not consistently connected with existing literature, particularly in areas related to physical activity. The authors must focus the discussion on presenting clear justifications for all the claims they make based on the statistical findings. The absence of a clear link with studies on physical activity, especially concerning older adults, limits the broader relevance of the findings, especially given the target population of the study. It is, therefore, important to contextualize the data by including studies on physical activity and cognitive impairment in the geriatric population. - Furthermore, the discussion does not provide concrete, actionable policy implications for this area, so the authors need to focus more on creating recommendations for the target population. | Thanks for the suggestions.   - We have revised the Discussions section accordingly. Regarding physical activity among older populations, we added that information with the reference (“Older people often engage in less physical activities due to retirement, health conditions, or reduced mobility, which then contribute to a positive energy balance and promote weight gain over time [37]) as it explains why OAO were more common among older groups. We revised the sentence and the reference. Page 16 lines 461-463. - For policy implication, we have added that information within paragraphs in the Discussion section after elaborating each of the main contributing factors. We also stated the policy implication for the overall findings in pages 17-18 lines 511-537. |
| - The manuscript also fails to meet PLOS ONE's data availability criteria. All the data should be provided as part of the manuscript or its supporting information, or deposited to a public repository, not just submitting the analysed and summarised ones. - Furthermore, the reference list requires significant revision to ensure all sources are updated and from within the last 10 years. Proper and appropriate citations are essential for the integrity of scientific publications. | - Many thanks for the comment on this. We have revised the statement according to the situation here. We were unable to make the dataset publicly available but it can be requested to the principal investigator of the study.   “In Indonesia, the regulation of data sharing agreements follows the rules by the Indonesian Ministry of Health Regulation (Permenkes) No. 85/2020, which pertains to the Transfer and Use of Materials, Information Content, and Data. Consequently, requests for the data underlying the study can be directed to the principal investigator of the 2018 Indonesian Food Barometer study, Dr. Helda Khusun.”   - We have updated the reference as suggested, and the citations. However, exceptions were made for these reference as they are the main reference/ guidelines and have not been updated/ replaced.   [19] WHO Expert Consultation. Appropriate body-mass index for Asian populations and its implications for policy and intervention strategies. Lancet Lond Engl. 2004 Jan 10;363(9403):157–63.  [20] O’Donnell O, van Doorslaer E, Wagstaff A, Lindelow M. Analyzing Health Equity Using Household Survey Data: A Guide to Techniques and their Implementation [Internet]. Washington, D.C.: The World Bank; 2008 [cited 2022 Dec 9]. Available from: <http://elibrary.worldbank.org/doi/book/10.1596/978-0-8213-6933-3>  [21] Wagstaff A. The bounds of the concentration index when the variable of interest is binary, with an application to immunization inequality. Health Econ. 2005 Apr;14(4):429–32. |
| While using an appropriate index, the statistical analysis needs to be applied more rigorously. This is particularly evident in the cross-sectional limitations and the need for detailed descriptions of the analysis. The conclusions, at present, are only partially supported by the data. Therefore, this manuscript needs a major revision. The authors should re-evaluate their methodology to provide a more robust justification for the statistical choices and address all limitations of cross-sectional data while providing well-detailed data analysis and interpretations. In addition, the discussion needs strengthening by linking it to existing literature, updating all the references, providing full data access, and providing actionable recommendations for future research and policy. Failure to address these significant concerns will prevent the manuscript from being considered for publication. | Many thanks for your constructive suggestions. We hope the revisions have addressed the concerns raised. |
| **Reviewer 3**  The topic is highly relevant and the research was very well conducted, but it is not new. It would be interesting if the authors could present suggestions for effective changes by governments and populations in order to reduce inequality and the prevalence of overweight and obesity. It is not enough to say that strategies are necessary; proposals must be presented in one or two paragraphs of the discussion, so that measures can be taken to find solutions. | Thank you for the suggestions.  We have added some information on the policy implications in the Discussions section as summarised in pages 17-18 lines 511-537. Before that part, we also integrated some suggestions for OAO interventions/programs within the Discussion section when explaining the role of the main contributing factors.  The Conclusions also included the suggested strategies. Page 19 lines 558-562. |
