# Supplementary material for: Socioeconomic inequalities in overweight and obesity among adults: Results from the Indonesian Food Barometer study
Source: PLoS One. 2025 Dec 5;20(12):e0337170. doi: 10.1371/journal.pone.0337170 (PMC12680161; doi:10.1371/journal.pone.0337170)
Supplement: Attachment — Submitted filename: r2 Response to Reviewers.docx [file pone.0337170.s004.docx]

| **Comments** | **Response**  [Pages and lines refer to the file ‘Manuscript’ or the clean version] |
| --- | --- |
| **Journal requirements** |  |
| 1. Please review your reference list to ensure that it is complete and correct. If you have cited papers that have been retracted, please include the rationale for doing so in the manuscript text, or remove these references and replace them with relevant current references. Any changes to the reference list should be mentioned in the rebuttal letter that accompanies your revised manuscript. If you need to cite a retracted article, indicate the article’s retracted status in the References list and also include a citation and full reference for the retraction notice. | We sincerely thank the editor for highlighting the importance of ensuring the accuracy and completeness of the reference list. We have thoroughly reviewed all references cited in the manuscript and confirm that none are retracted. Therefore, no references required removal or replacement due to retraction.  In response to Reviewer 2’s comment (comment no. 4), we have added four new references (28, 29, 38, and 39) to strengthen the manuscript. These additions have been carefully selected and verified to maintain the integrity and relevance of the work.  The newly added references are as follows:  28. Trübswasser U, Verstraeten R, Salm L, Holdsworth M, Baye K, Booth A, Feskens EJM, Gillespie S, Talsma EF. Factors influencing obesogenic behaviours of adolescent girls and women in low‐ and middle‐income countries: A qualitative evidence synthesis. Obes Rev [Internet]. 2021 Apr [cited 2025 Aug 3];22(4). Available from: https://onlinelibrary.wiley.com/doi/10.1111/obr.13163  29. Alvarez-Saavedra V, Levasseur P, Seetahul S. The Role of Gender Inequality in the Obesity Epidemic: A Case Study from India. J Dev Stud. 2023 July 3;59(7):980–96.  38. Pu F, Lin J, Wei Y, Li J, Liao X, Shi L, Zeng X, Hu W. Association of dietary behavior patterns of middle-aged and older adults with their obesity metabolic phenotype: a cross-sectional study. BMC Public Health [Internet]. 2024 Aug 26 [cited 2025 Aug 3];24(1). Available from: https://bmcpublichealth.biomedcentral.com/articles/10.1186/s12889-024-19781-3  39. Dewi S, Setyowati S, Rachmawati IN, Ayubi D. Understanding Minangkabau Culture and its Impact on Weight Management Strategies in Overweight and Obese Women: A Qualitative Study. Open Public Health J [Internet]. 2024 Apr 26 [cited 2025 Aug 3];17(1). Available from: https://openpublichealthjournal.com/VOLUME/17/ELOCATOR/e18749445300276/ |
| **Reviewer #2**  Dear Authors, Thank you for submitting your revised manuscript. This study addresses a highly relevant public health issue in Indonesia, examining the complex interplay between socioeconomic status (wealth and education) and overweight/obesity (OAO). The application of the Wagstaff normalized concentration index and decomposition analysis provides valuable quantitative insights into the nature and drivers of these inequalities, contributing significantly to the literature in this area, particularly within the Indonesian context. The manuscript is generally well-written and methodologically sound. | We sincerely thank Reviewer #2 for the positive and encouraging remarks on our revised manuscript. We appreciate your recognition of the study’s relevance, the methodological rigor applied through the Wagstaff normalized concentration index and decomposition analysis, as well as the contribution our findings make to understanding socioeconomic inequalities in overweight and obesity within Indonesia. Your feedback is highly motivating and valuable for strengthening our work. |
| 1. Crucial Clarification of Data Collection Period: There remains significant ambiguity in the Methods section (Lines 77-78) regarding the data collection dates. The text states, "The 2018 Indonesian Food Barometer (IFB) survey collected data from March 2009 to January 2010". This wording is confusing and requires immediate correction for clarity. Please explicitly clarify whether the analysis uses data from a survey fielded in 2018, or if it uses data collected during 2009-2010 as part of a survey labeled or reported in 2018. If the data are indeed from 2009-2010, the manuscript framing needs adjustment, and the timeliness/relevance requires stronger justification given the rapid nutrition transition since that period. | Thank you for highlighting the ambiguity regarding the data collection period. We apologize for the confusion caused by the phrasing in Lines 77-78 in the previous manuscript.  To clarify, the analysis is based on data collected as part of the "2018 Indonesian Food Barometer (IFB)" study; however, the fieldwork was actually conducted from December 2017 to March 2018. The reference to 2009-2010 in the manuscript was an error.  We have revised the Methods section to state explicitly:  "The Indonesian Food Barometer (IFB) survey collected data from December 2017 to March 2018 in six provinces …." –Lines 75-77. |
| 2.Data Availability Statement: Your manuscript states data access is restricted due to institutional/governmental policy and available upon request from SEAMEO RECFON. PLOS ONE's policy requires unrestricted access unless an exception is approved. "Available upon request" is generally insufficient. Please work directly with the PLOS ONE editorial office to validate this exception. This requires providing verifiable details of the restricting policy and ensuring the SEAMEO RECFON access route is confirmed as compliant with the journal's exception standards. This policy point must be formally resolved for the manuscript to proceed. | Thank you for your comment regarding the Data Availability Statement.  Due to national institutional and governmental policies protecting participant confidentiality, access to the raw individual-level data from the 2018 Indonesian Food Barometer study is restricted and cannot be openly shared. Only data provisioned and approved under institutional data use agreements through SEAMEO RECFON can be accessed.  To ensure transparency and reproducibility, we have included detailed summary data and completeness information for all analysed variables in Supplementary Table S1, which fully supports the findings reported in the manuscript.  Researchers interested in accessing the raw dataset may request permission directly from SEAMEO RECFON, subject to institutional approvals. We have contacted the PLOS ONE editorial office to confirm that this data sharing arrangement meets the journal’s data availability standards and await further guidance.  We remain committed to maximizing data transparency while adhering to ethical and legal standards governing these sensitive data.  We mentioned Table S1 in the Methods: “We excluded pregnant women and respondents with missing information, yielding a sample of 1,604 cases (see Supplementary Table S1 for detailed sample characteristics and data completeness).” – Line 86 |
| 3. Role of Initial Logistic Regression: The manuscript briefly mentions performing logistic regression "before undertaking the inequality analysis" (Line 117). The precise purpose and how the results of this initial analysis informed the primary inequality analysis remain slightly unclear. Please briefly clarify this connection or rephrase to avoid potential reader confusion about the role of this preliminary step. | Thank you for bringing attention to the need for clarification regarding the role of the initial logistic regression analysis.  We have revised the Methods section to clarify that the logistic regression was conducted to identify and assess the association between socioeconomic and demographic variables and overweight and obesity (OAO) among Indonesian adults. This step provided an initial understanding of the covariates relevant to OAO and informed the selection of variables to be included in the subsequent inequality analysis. The regression ensured that the factors decomposed in the Wagstaff concentration index were empirically relevant and statistically associated with OAO within this study population.  The revised text now reads:  “Prior to the inequality analysis, we performed logistic regression to identify factors significantly associated with OAO. The results informed the selection of covariates included in the decomposition of the concentration index.” –Lines 116-118.  This change clarifies the analytical sequence and the rationale for the preliminary regression, improving overall transparency in the methodological approach. |
| 4. Discussion Nuance: The discussion is solid but could benefit from slightly deeper interpretation in places. For example, the finding that significant wealth and education inequalities were observed only among males is interesting. Could you offer slightly more developed speculation, citing relevant literature on potential contributing factors specific to Indonesia (e.g., gender roles, labor market participation)? A more detailed exploration of why certain factors (like age and marital status) had large negative contributions in the decomposition analysis could also add valuable nuance. | Thank you for your insightful comment. We have expanded the Discussion to better explain why significant socioeconomic inequalities in OAO by wealth and education were observed only among males. We now highlight how gender roles, labor participation, and sociocultural factors in Indonesia contribute to these patterns, including differences in household responsibilities for women and occupational exposures for men (Lines 230-239; 305-308).  We also clarified the negative contributions of age and marital status in the decomposition analysis, with references to relevant literature supporting these interpretations. These revisions provide a more nuanced understanding of the gendered and demographic dynamics of socioeconomic inequality in OAO in Indonesia (Lines 323-333). |
| 5. Reference Recency: While generally good, please perform a final check on references cited for broad background information (e.g., global statistics) to ensure the most current sources are used, especially if older than 10 years. Foundational methodological citations are exempt. | Regarding the request to ensure the currency of references for broad background information, especially those older than 10 years: we conducted a comprehensive audit of these references. Foundational methodological sources were retained. For general epidemiological data and global statistics, we updated or reaffirmed the use of the latest, most relevant sources (e.g., the 2024 WHO fact sheet on obesity and overweight was replaced with the 2025 version). Outdated references have been replaced or removed as appropriate to ensure up-to-date contextual accuracy. |
| 6. Minor Wording Consistency: Please ensure consistent terminology throughout, for example, using "had OAO" consistently instead of variations like "lived with OAO". | Thank you for highlighting the importance of consistent and respectful terminology. Based on current international best practices and terminology preferences reflected in recent PLOS ONE publications and related systematic reviews, we have carefully reviewed and revised the manuscript to consistently use people-first terms such as “people living with overweight and obesity (OAO)” throughout. We have removed expressions such as “lived with OAO” and “overweight and obese people” to minimize stigma, promote respectful academic writing, and align with the language norms of PLOS ONE and the wider scientific community. |
| Reviewer #3  The authors complied with all my requests, and the modifications resulting from the requests of the other reviewers substantially improved the quality of the manuscript, so I consider that it should be accepted for publication. | We sincerely thank Reviewer #3 for the positive evaluation and encouraging remarks regarding our manuscript. We appreciate the recognition of the improvements made in response to the reviewers’ comments. We are grateful for the opportunity to contribute to the literature on socioeconomic inequalities in overweight and obesity among Indonesian adults and look forward to the publication of our work. |
